# Towards cataloguing and characterising advance care planning and end-of-life care resources

**DOI:** 10.1186/s12904-022-01102-3

**Published:** 2022-11-29

**Authors:** Edric Aram Ramirez-Valdez, Clare Leong, Frances Wu, Sarah Ball, Giulia Maistrello, Graham Martin, Zoë Fritz

**Affiliations:** 1 School of Clinical Medicine, University of Cambridge, Addenbrooke’s Hospital, Hills Rd, Cambridge, CB2 0SP UK; 2 The Healthcare Improvement Studies Institute, THIS Institute, University of Cambridge, Cambridge Biomedical Campus, Clifford Allbutt Building, Cambridge, CB2 0AH UK; 3 RAND Europe, Cambridge, UK

**Keywords:** Advance care planning, Terminal care, Communication, Clinical decisions

## Abstract

**Background:**

Resources for healthcare professionals, patients and those important to them relating to planning and coordinating treatment and care at the end of life are abundant, and can be difficult to navigate. However, they have not been systematically collated or catalogued in terms of their purpose, scope or intended audience.

**Aim:**

To collate, categorise and characterise advance care planning and end-of-life treatment and care (EoLT + C) resources directed towards healthcare professionals, patients and their families.

**Methods:**

Rapid review and thematic synthesis of resources available in the United Kingdom. Google searches and reviews of websites belonging to selected organisations that develop and publish materials relating to EoLT + C, and advance care planning were used. Materials were included if they were intended for those over 18 living in the UK and pertained to five domains of EoLT + C: identifying those approaching end of life; accessing EoLT + C services; conducting important conversations about EoLT + C and preferences; advance care planning, including recording of preferences and plans; and ensuring that plans and preferences are accessed and used by health and social care services.

**Results:**

246 resources directed at healthcare professionals, patients and their families were identified, collated, catalogued and made internationally available for clinicians, researchers, patients and the public. 61 were classified as interactive, providing decision support in EoLT + C that went beyond simply providing information. Of these, there was notable content overlap among tools for identifying patients in their last year of life. There was variation in the development of tools across all domains of end-of-life care by geography and patient group. Few interactive resources integrated seamlessly with a digital interface or healthcare provider workflows. Incentives for the adoption of best-practice appeared rare.

**Conclusions:**

We present a repeatable and scalable approach to the cataloguing and characterisation of palliative care resources. The identified resources will be of benefit not only to those in the UK but to those in other countries, developing or evaluating their own resources for aiding professionals and patients to plan and deliver excellent treatment and care at the end of life.

**Supplementary Information:**

The online version contains supplementary material available at 10.1186/s12904-022-01102-3.

## Background

The planning, coordination and delivery of high-quality, individualised care for patients approaching the end of life is a complex process, at what can be an extremely challenging time for patients and those important to them. Current guidance from the UK National Institute for Health and Care Excellence (NICE) in England highlights key aspects of the process, including good practice in identifying adults who may be approaching the end of their life, advance care planning, and communicating and sharing information between services [1].

Although the guidance is comprehensive in scope, it focuses broadly on general statements and recommendations regarding the delivery of services relating to end-of-life care, rather than specific advice about how these should be achieved. Furthermore, evidence suggests that implementation of these guidelines in UK health practice is limited. The second round 2019/20 National Audit of Care at the End of Life found that 36% of dying patients did not have an individualised end-of-life care plan in their medical records [2], and that 11% of deceased patients had not been given the opportunity to discuss dying before their death. These implementation problems can lead to poorer and less personalised care for individuals at the end of life; to unsatisfactory experiences for carers; and to people either not receiving the care they need, or receiving unnecessary or inappropriate interventions that they would prefer not to have and that are costly for health systems [3–5].

A multitude of resources exists to support both healthcare providers and patients and those important to them in navigating the complex landscape of end-of-life care. Examples include tools to help identify those who may be approaching the end of their life, and proformas to support clinicians and patients to undertake advance care planning. Resources of this kind have the potential to bridge the gap between espoused recommendations and day-to-day practice, for example by making recommendations accessible to various audiences who might make use of them. However, collation and critical analysis of these resources, their use and impact is scarce in the published literature. No comprehensive catalogue of available resources designed to improve treatment and care at the end of life currently exists; efforts to consolidate existing resources in the literature have largely been restricted to those focussing on specific disease areas (e.g. COVID-19) [6] and/or patient populations [7]. In the absence of a systematic typology of these resources, both the degree of duplication (multiple resources attempting to solve the same problem) and the areas of unmet need (processes or areas for which supporting resources do not yet exist) remain unclear.

Understanding the full landscape of resources available to healthcare providers, patients and those important to them is a necessary first step both in ensuring that effective resources are shared and implemented more widely (both locally and internationally), and in highlighting areas where the creation of additional resources could provide benefit.

We aimed to:

1. Systematically identify and catalogue available resources surrounding end-of-life care and advance care planning for healthcare professionals, patients and those important to them (such as family members and informal carers).
2. Identify the resources that contain an element of interactivity for the user and would be used in the practice of planning and delivering care.
3. Identify characteristics of resources likely to be most helpful in embedding good practice in end-of-life care.

## Methods

We used an Internet search engine (Google) and prior knowledge of organisations working in this field to identify available resources between June and September 2021, which we then collated and categorised by obtaining resources, summarising their key characteristics and comparing their features. We define “resource” to include any materials aimed at providing information about end-of-life care or supporting the processes involved, including, but not limited to: booklets, webpages, videos, podcasts, tools/toolkits, or fact sheets.

### Inclusion criteria

Resources designed to support end-of-life care processes and advance care planning for adult patients (aged 18 or over), who were either identified as likely to be in the last year of life, or planning ahead for this period (e.g. due to life-limiting conditions) were included. We included resources aimed at health and social care professionals, individuals considering end of life, and those important to them. Included resources were intended for a UK audience or international resources with relevance to a UK audience. A resource produced outside of the UK was deemed relevant for a UK audience if it included references to the NHS or UK regulatory or clinical bodies (e.g. NICE, General Medical Council, Royal College of Physicians).

### Exclusion criteria

Resources that had no relevance for a UK audience, or were not focused on medical preferences, treatment or care (e.g. resources relating to financial matters or environmental factors such as music to be played during last days of life) were excluded.

### Search and selection strategy

First, resources were identified using the websites of organisations working in this area familiar to research team members, searching for content of relevance to end-of-life care. Resources that were accessible via those links were then considered for inclusion in the catalogue.

To maximise coverage of relevant materials from the selected organisations, a systematic search approach was taken which incorporated exploring each organisation’s website using Google searches of key terms ("advance care" OR "end of life" OR “palliative”) appended to a website identifier. As an example, the Google search entry for Age UK website would be expressed as *site:ageuk.org.uk "advance care" OR "end of life" OR “palliative”*. The top 20 results were reviewed to determine whether the material accessed met inclusion criteria and whether it had been previously captured in the catalogue. The cut-off of 20 results for a single organisation’s website was selected because patients and physicians are unlikely to search through more than 2 pages of search results when they use a search engine; as many as 92% of search traffic hits are derived from the first page of Google results [8].

To identify resources beyond the research team organisation list, a systematic approach was carried out using a series of key search terms on the Google search engine (see Additional file 1). The top 50 results were reviewed to determine whether the material met inclusion criteria. The cut-off of 50 results was intended to maximise relevant catalogue additions, given that this search did not specify a publishing organisation.

Finally, for resources that comprised lists of other resources, a click-through of each link to search for additional tools/toolkits was carried out. Where a tool/toolkit or proforma was identified, it was also added to the catalogue.

### Data extraction and synthesis

Resources that met the inclusion criteria were collated into a spreadsheet. Data was extracted from each resource regarding key characteristics – e.g. organisation, resource modality, reference to evidence etc. (see Table 1). Codes for each characteristic were initially developed inductively through review of the first 100 resources, and the coding frame was then applied to remaining resources. Coding was reviewed upon completion; disagreements were discussed and coding was adapted as necessary. In addition, we sought to categorise each resource in terms of its relevance to the following domains in the process of planning and delivering care for people approaching end of life:

1. Identification of people who may be entering the last year of life
2. Access to end-of-life care services
3. Initiation and conduct of important conversations about end-of-life care and preferences
4. Advance care planning, including recording of preferences and plans
5. Ensuring that plans and preferences are accessed and used by health and social care services

**Table 1:** Key features collated for each resource. Categorisations with asterisks had over 100 unique labels

Data Field	Categorisations Assigned
Resource title	• Title*
Publishing Organisation	• Organisation name*
Organisation type (e.g. charity)	• Charity • Hospice • Not for Profit/Non-Governmental Organisation • Private Sector • Professional Membership Organisation • Public Sector (Local Authority) • Public Sector (NHS) • Regulatory/policy • Research Institute • Web/Information Hub
Modality	• Booklet • Fact Sheet • Podcast • Tool/Toolkit • Video • Web Page
Function	• Informational • Learning and development • List of Resources • Proforma / Documentation • Guidelines / Standards for Practice
Link	• Hyperlink to resource*
Target audience	• Caregivers, family, and important others • General • Health and social care professionals • Patients and people approaching end of life
Target condition/population (e.g. cancer)	• Acute care • Advanced Cancer • Alzheimer's • Brain tumour • Cancer • Cancer and non-cancer patients • Care home residents • COVID19 • Dementia • Diabetes • Frailty • General • Heart Failure • Homeless people • Intellectual disabilities • Kidney disease • Learning disabilities • Learning disabilities and Covid • LGBT + • Life limiting illness and COVID19 • Long term lung condition • Motor neurone disease • Pancreatic cancer • Parkinson's Disease • South Asian community
Population specificity	• Disease-specific • General • Patient community • Setting
Country setting (if not UK)	• Ireland • Netherlands • USA
1.Identification of people who may be entering the last year of life	• Yes • No
2. Access to end of life care services	• Yes • No
3. Initiation and conduct of important conversations about end-of-life care and preferences	• Yes • No
4. Advance care planning, including recording of preferences and plans	• Yes • No
5. Ensuring that plans and preferences are accessed and used by health and social care services	• Yes • No
Refers to evidence / literature	• Yes • No
Refers to NICE guidelines?	• Yes • No

### Interactive resources for thematic characterisation

We then sought to identify those resources within the catalogue that included ‘interactive’ elements, defined by the following criteria:

- Any tools, toolkits or proformas which facilitated decision-making through interactivity with the user (e.g. decision support tool), included instruction for the user (e.g. algorithm on what to do when) or were intended to be used as part of the planning and delivery of care (e.g. proforma) were categorised as interactive resources.
- Resources and resource lists which had no interactivity, instruction, simplification or direction were categorised as non-interactive.

Two researchers independently reviewed each resource and allocated it to either the interactive or non-interactive group, on the basis of these criteria. Where the reviewers were in disagreement, allocation was determined via discussion with a third researcher.

In order to identify the characteristics which might be most helpful in embedding best practice end-of-life care, interactive resources were then explored individually and sequentially across each of the five domains described above. One researcher reviewed the content of interactive resources line-by-line, and inductively identified themes arising relating to their utility, which could influence adoption of best practice. These themes were discussed and agreed with other team members, and are presented below under the domains of planning for end-of-life treatment and care to which they related.

## Results

### Resource catalogue

**Fig. 1:**
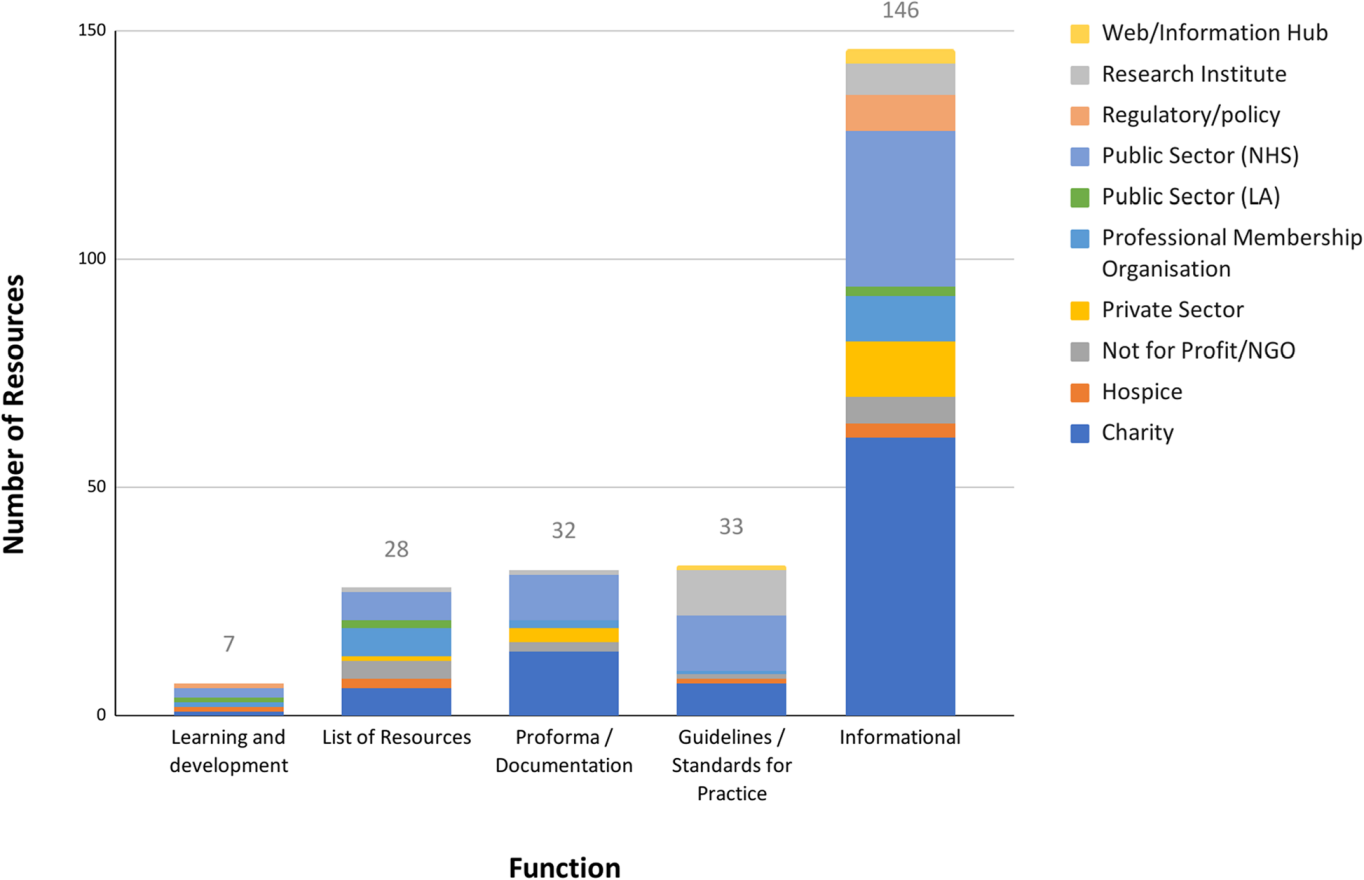
Resources by function and organisation type

The search yielded 246 resources (see Additional file 2 for link to complete catalogue), which served different functions. Each resource was categorised into one of five groups, based on our assessment of its primary function: purely informational, guidelines on standards for practice, proformas (document with space to be filled by user), resource lists or learning & development resources used for professional training and education. The resources were published by a range of organisations including research institutes, charities and UK National Health Service (NHS) organisations from hospital trust to national-level bodies (Fig. 1). The majority of resources (146/246, 59%) served as purely informational across the domains of end-of-life care. Across the various functions, resources were published by care delivery organisations, charities, research and regulatory bodies, as well as others, but most commonly by the public sector (NHS) and charities.

**Fig. 2:**
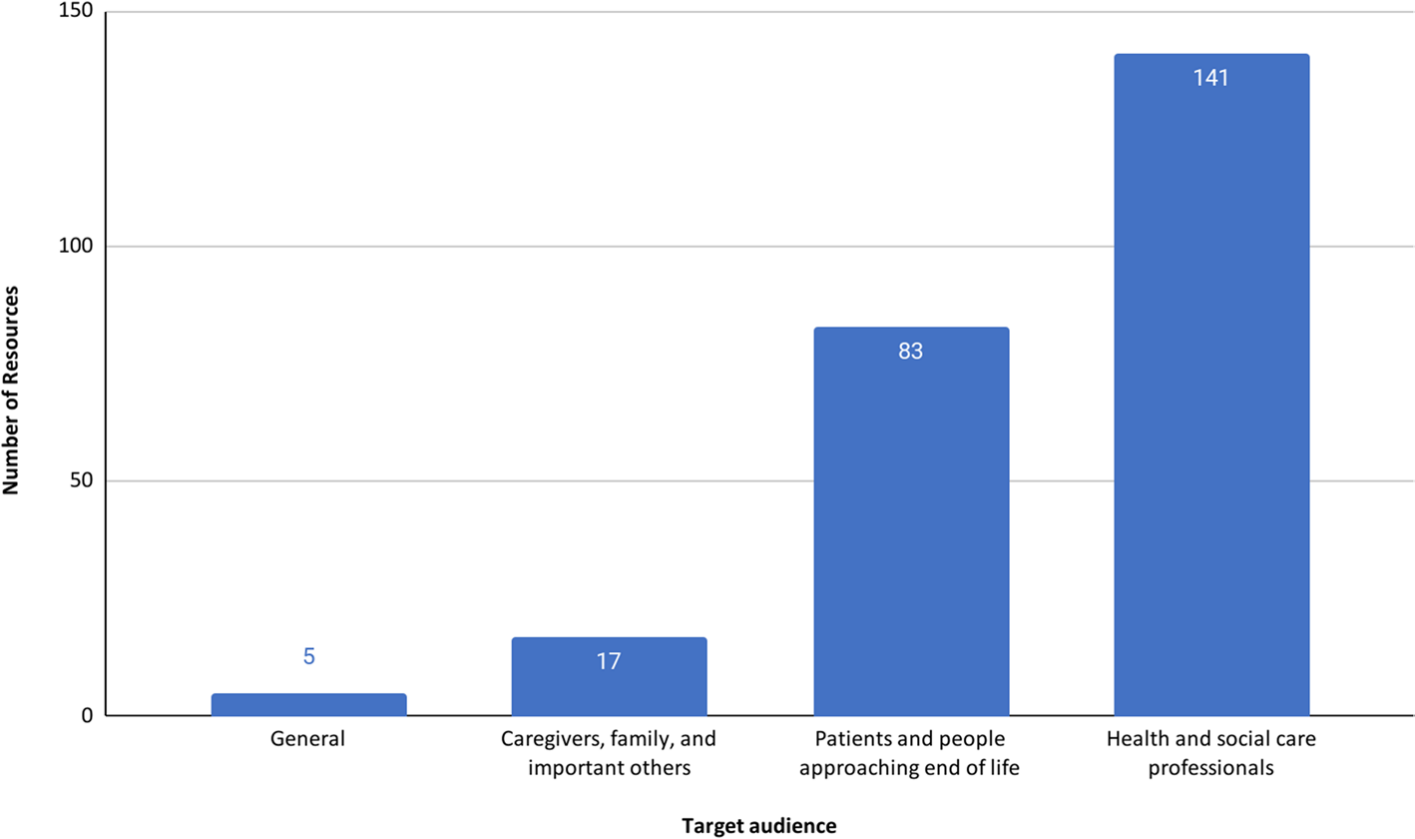
Resources by target audience

The resources were primarily directed towards healthcare professionals (141/246, 57%) and patients approaching the end of life (83/246, 34%) (Fig. 2).

**Fig. 3:**
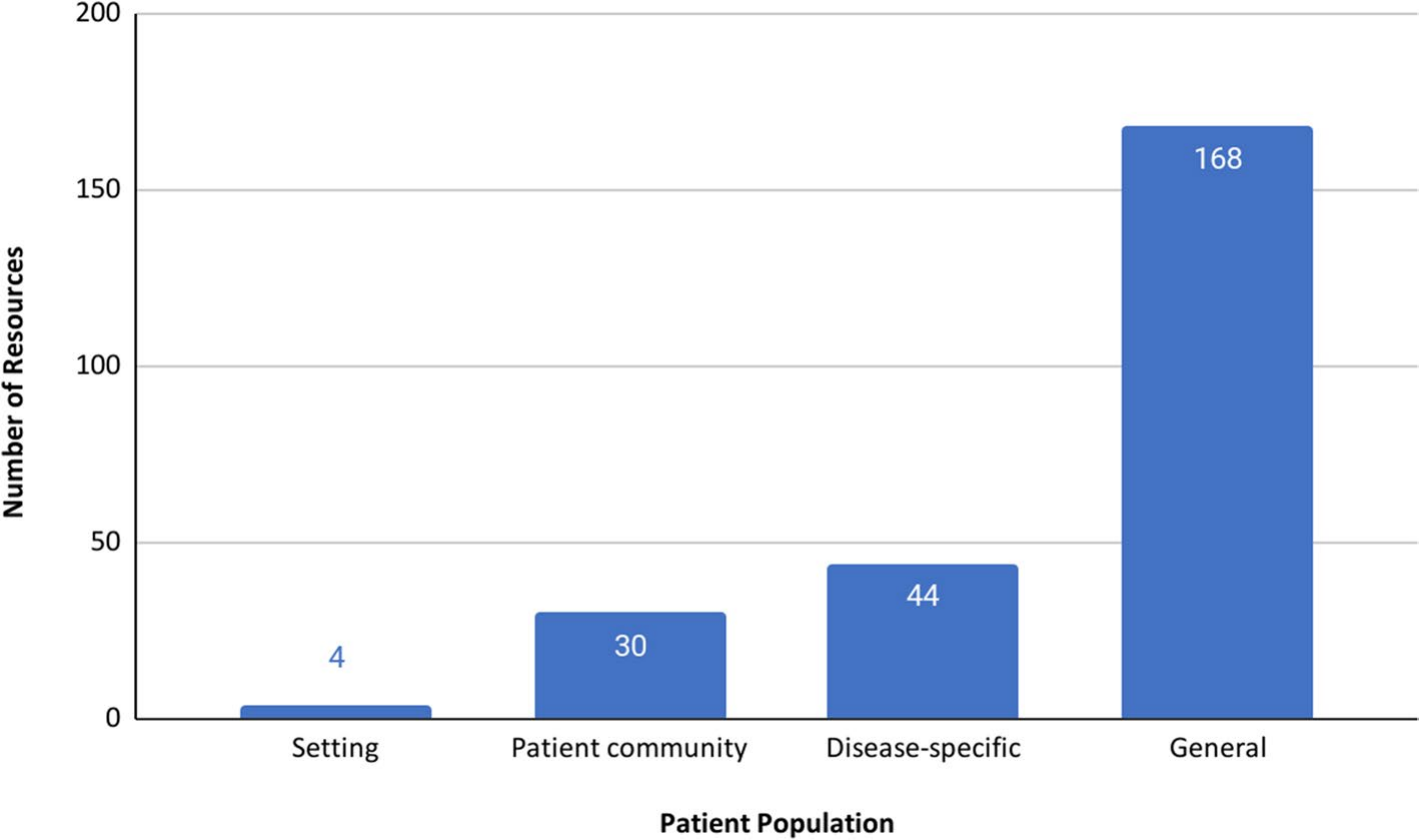
Resources by population addressed

The majority of resources (168/246, 68%) were not specific to any particular patient population (Fig. 3). Disease-specific, community-specific (e.g. homeless, learning disabilities, LGBT +), and setting-specific (e.g. acute care, care homes) resources made up less than one third (78/246, 32%) of the resources.

**Fig. 4:**
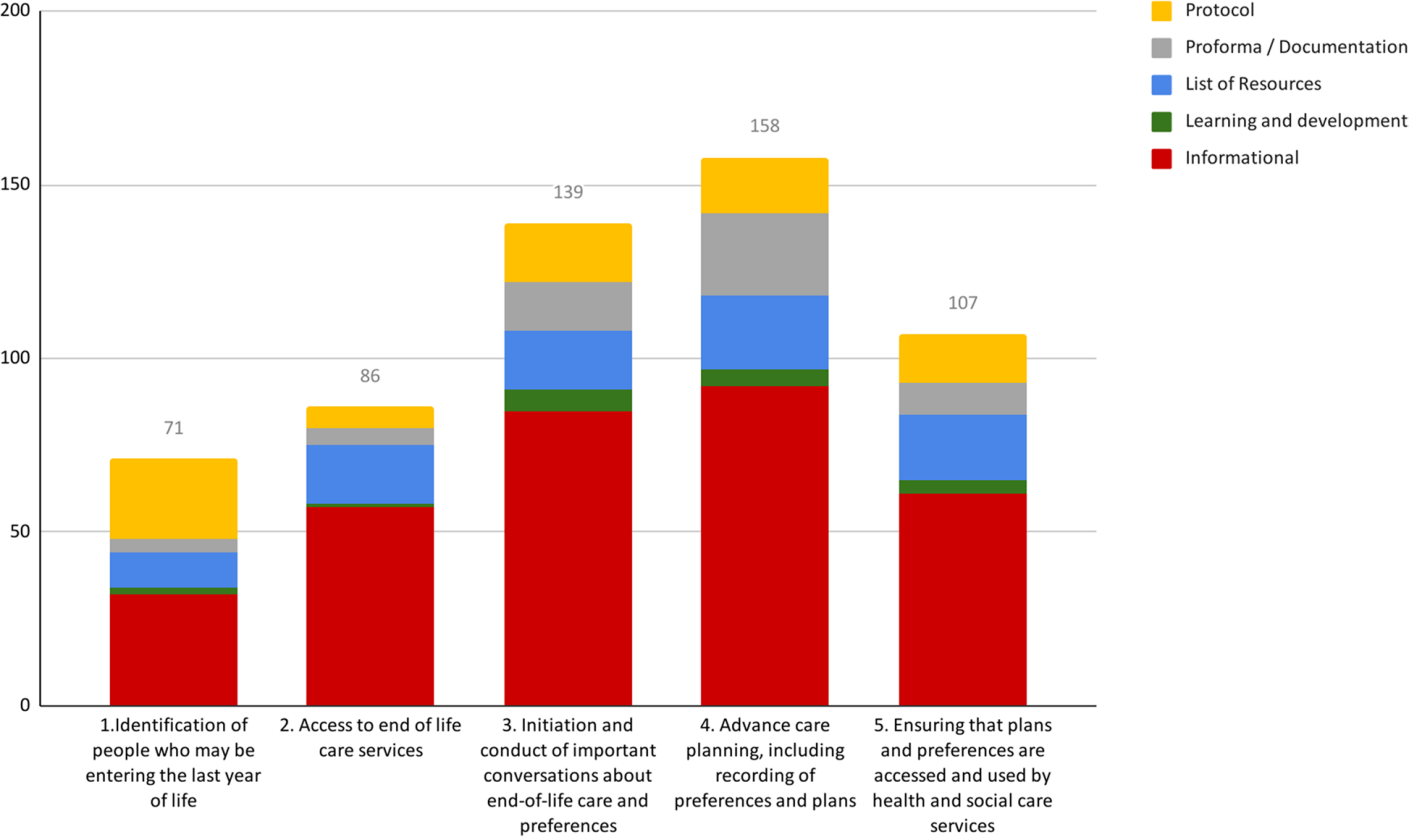
Resources by function and relevance to each domain in the process of end-of-life care

Of the five domains in the process of planning and coordinating end-of-life treatment and care we identified, advance care planning was addressed most frequently in the resources (158/246, 64%), followed by initiation and conduct of important conversations (139/246, 57%) (Fig. 4).

### Interactive resources

Sixty-one of the 246 resources (25%) were classed as interactive, and subjected to further characterisation.

**Fig. 5:**
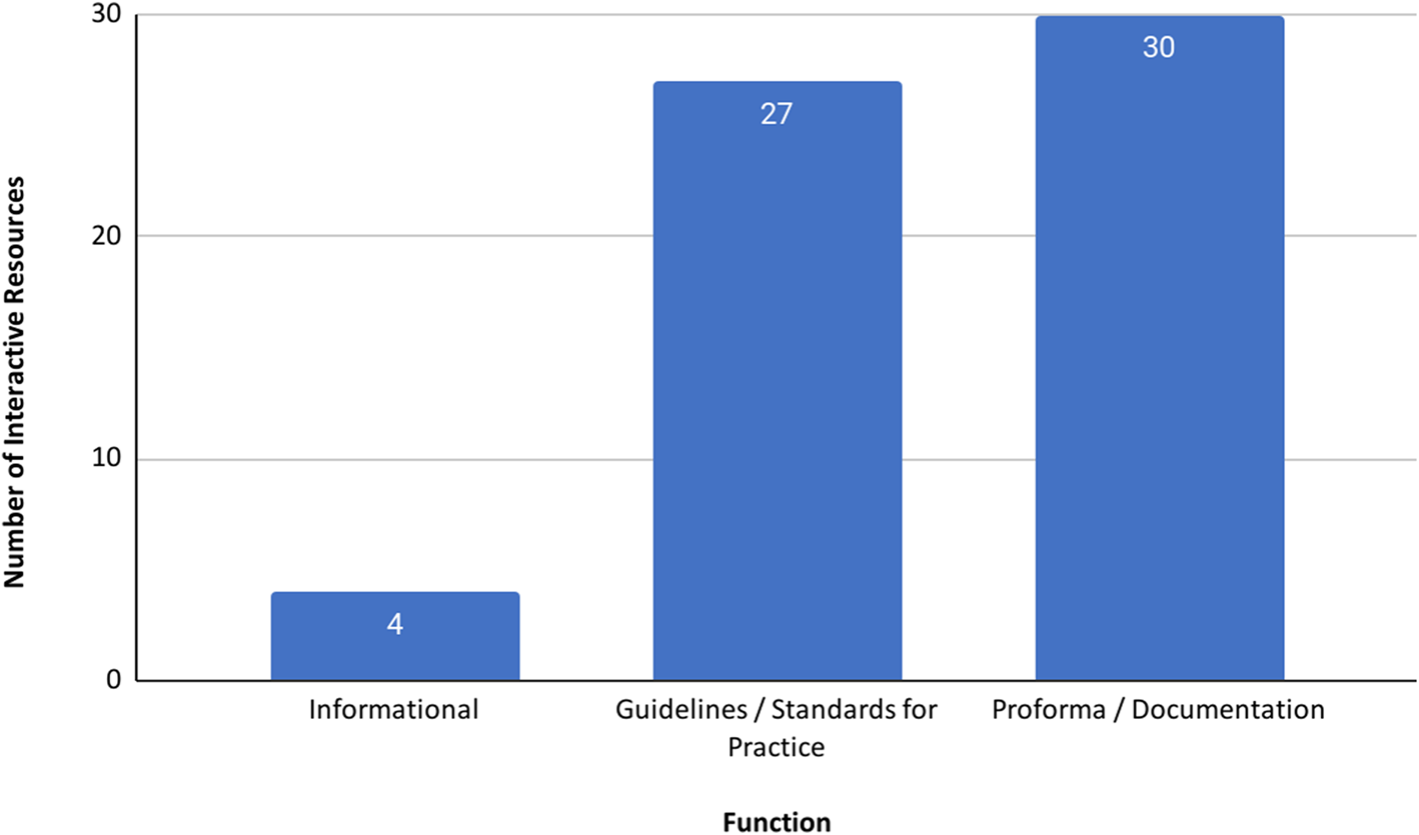
Interactive resources by function

In contrast to the full catalogue, the interactive resources were predominantly proformas and standards for practice, with only four of the 61 interactive resources being informational (e.g. CQC End of life care ratings map based on user’s home address) [9] (Fig. 5).

**Fig. 6:**
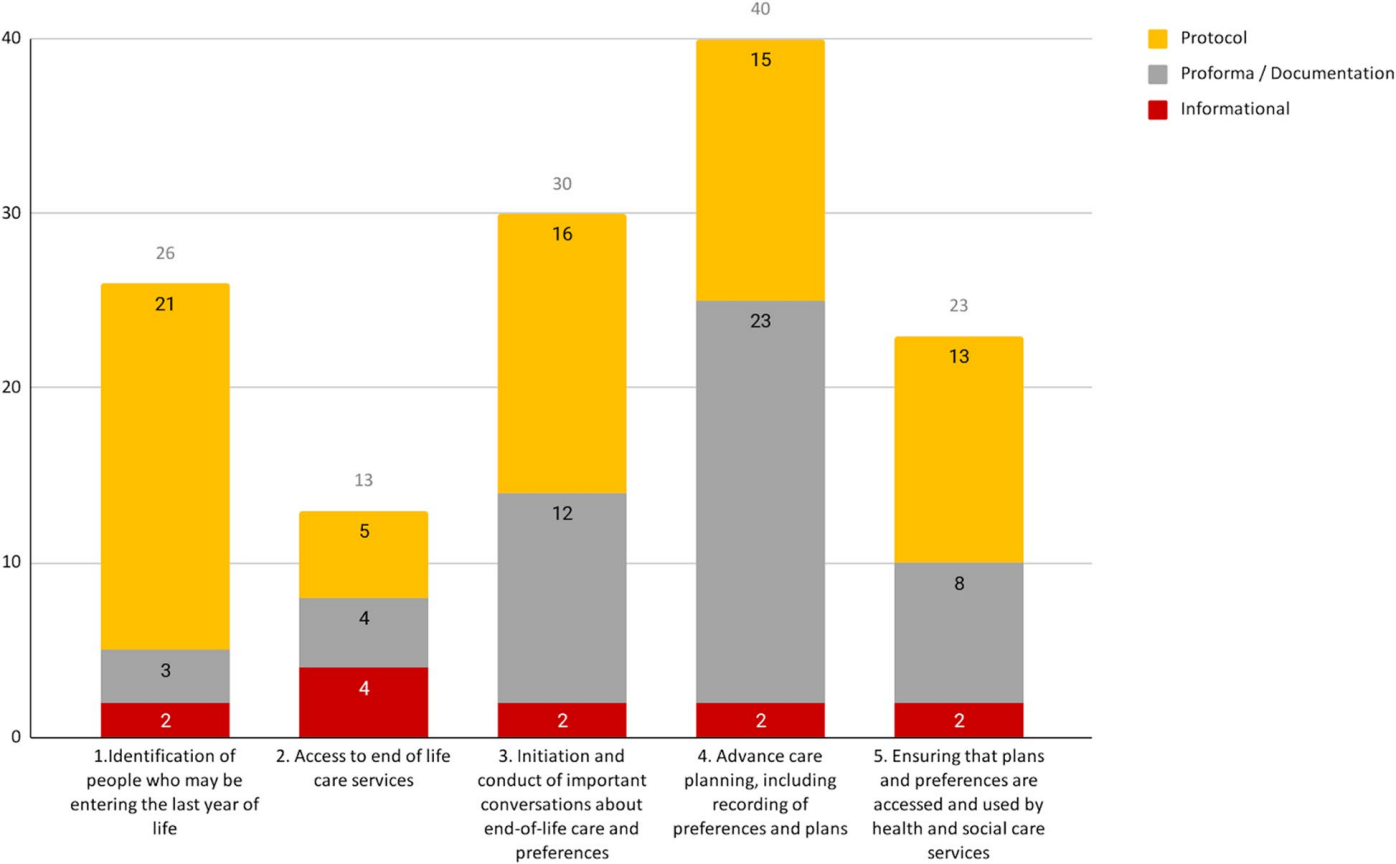
Interactive resources by function and relevance to each domain of end-of-life care

Again, the most frequently addressed domain was advance care planning (40/61, 66%) (Fig. 6). Access to end-of-life care services was covered by only 13 interactive resources (21%). We therefore focused our thematic characterisation on the other four domains, where more material was available.

### Identification of people who may be entering the last year of life

Choice and duplication in tools for identifying patients in their last year of life: twenty-six interactive resources pertained to the identification of patients in their last year of life. Many of these tools make reference to or incorporate the Gold Standards Framework (GSF) and/or the Amber Care Bundle, which are both mentioned in NICE guidance for identifying patients nearing their end of life [1, 10, 11]. These tools, along with the Supportive and Palliative Care Indicators Tool (SPICT) can be used for general populations, while other tools such as the Liver Map and PiPS-B prognosticator are disease-specific guides for identifying patients in their last year of life [12–14].

A comparator resource exists for *identifying* patients, but not other domains of end-of-life care and treatment: Healthcare Improvement Scotland has produced a ​​Palliative Care Identification Tools Comparator which compares 13 tools for identifying patients in their last year of life [15]. No tools were identified that compare resources for other domains of the end-of-life care journey (e.g. advance care planning including recording of preferences and plans, and initiating and conducting important conversations about end-of-life care preferences).

### Initiation and conduct of important conversations about end-of-life care and preferences

Communication conduct and content are framework- and context-dependent: thirty interactive resources addressed the topic of initiating and conducting important conversations about end-of-life care and preferences. There was notable variation between them in the ways they recommended conversations be conducted. For example, the REDMAP conversational framework, developed by Healthcare Improvement Scotland, outlines a step-by-step conversational prompt to establish rapport, and encourages a patient to begin the process of advance care planning [16]. In contrast, Pathway has an activity worksheet for homeless-sector health and social care workers to carry out an introspective exploration and planning ahead exercise to ensure the conversation they initiate with the patient is a successful one [17]. The ReSPECT process is advocated for the general population, with increasing relevance for those who are approaching the end of life; in contrast the NHS South of England End of Life Care (EoLC) tool-kit for care home staff uses a traffic light system to specify which types of conversations need to happen depending on a resident’s condition [18, 19]. In part, this variation in approach and content appears to relate to differences in the needs and wishes of the intended audience, and particularly the patient group concerned. However, the inconsistency may also reflect uncertainty, and an underdeveloped evidence base, about which approach is preferable for those involved.

### Advance care planning, including recording of preferences and plans

**Fig. 7:**
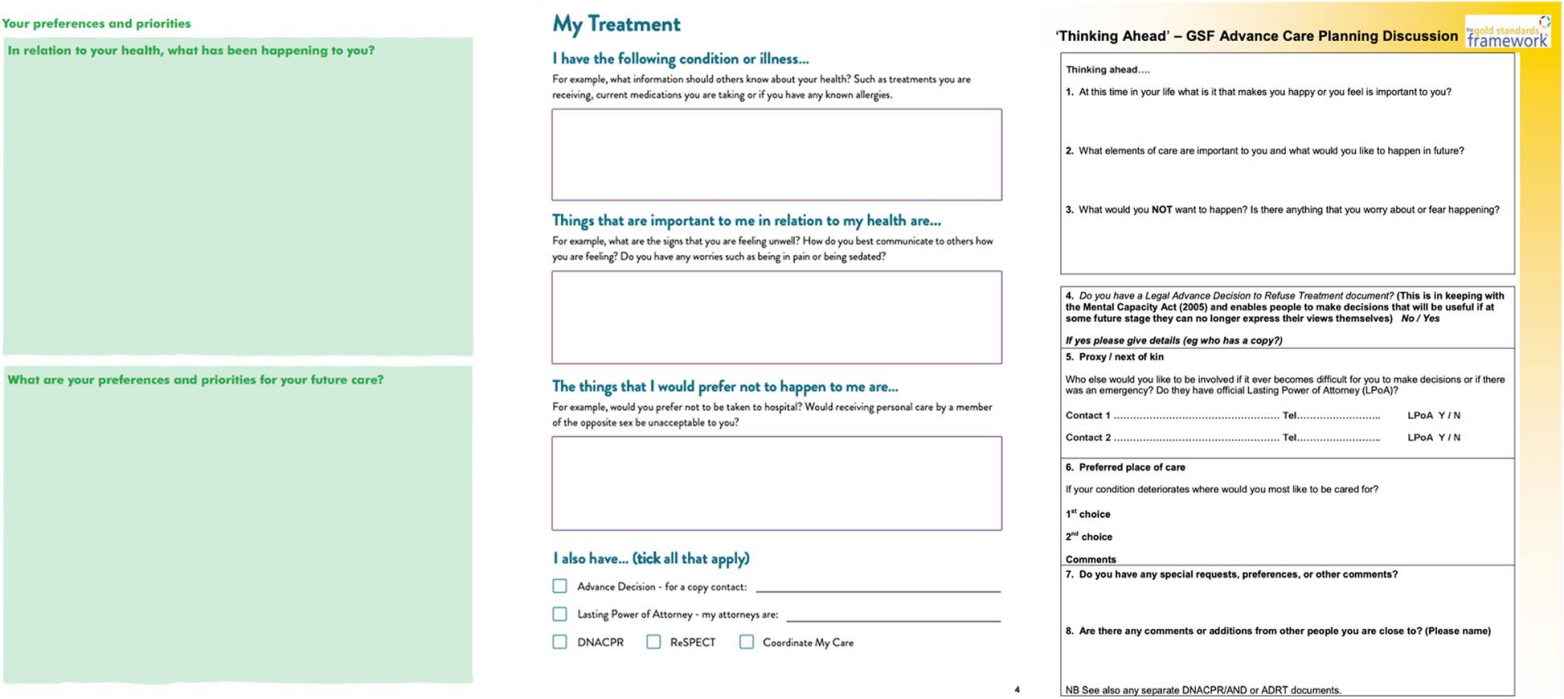
Sections of Macmillan Preferred Priorities for Care (left), Compassion in Dying Advance Statement (middle) and Gold Standard Framework: Thinking Ahead (right) proformas relating to advance care planning

Interactive resources for advance care planning are numerous and highly variable: forty interactive resources related to the “advance care planning, including recording of preferences and plans'' domain. Several were proformas permitting the individual, family members and healthcare providers to record various aspects of their healthcare preferences. These resources vary in format, wording and contextualisation (Fig. 7). For example, the Macmillan Preferred Priorities for Care document has fewer questions and provides less direction than the Compassion in Dying Advance Statement, despite having the same objective [20, 21]. Both have a different format from the Gold Standard Framework: Thinking Ahead document [22]. Again, such inconsistencies may reflect uncertainty about best practice. Cross-referencing between resources is abundant; the Compassion in Dying Advance Statement references ReSPECT, Coordinate My Care, and DNACPR as possible additional advance care planning documentation.

Below the national level, resources for advance care planning were also identified at varying geographic levels such as regions (NHS South of England), and devolved nations (Scottish Government and Healthcare Improvement Scotland). Advance care planning also seemed to be a major domain for the development of digital tools, such as My Decisions and My Wishes, intended to improve the patient experience of documenting their advance care plan [23, 24]. Potentially beneficial features of these digital tools include remote accessibility, the ability to digitally save progress when writing documents, and the integration of other important end-of-life activities such as last will and testaments, goodbye messages/videos, and funeral wishes.

### Ensuring that plans and preferences are accessed and used by health and social care services

Few interactive resources integrate seamlessly with healthcare provider workflows: the majority of interactive resources were paper-based proformas and thus were not functionally integrated into health and social care service workflows. Some digital tools, such as the EARLY Identification and Personalised Care Planning Toolkit, are designed to work with clinician electronic health record systems in primary and secondary care, such as EMIS and SystmOne [25]. The EARLY search tool also integrates guidance from SPICT and the Gold Standards Framework without the need for direct access to those documents.

Other digital tools such as My Decisions and My Wishes enable sharing of a patient’s advance care plan but require physical delivery of hardcopy documents to their physician. A manual tool designed to ensure the patient’s preferences and plans are accessed and used is the Advance Care Plan Passport—a wallet-sized document providing details of an individual’s advance care plan [26].

Incentives for adoption are uncommon: we found little evidence of the use of extrinsic incentives to encourage uptake of the interactive tools. One exception was the Daffodil standards—a set of best-practice recommendations for use in primary care recognised by the Care Quality Commission (CQC), which adopting GP practices are permitted to display publicly for patients to see [27].

## Discussion

### Resource catalogue

We have produced a catalogue of resources for treatment and care at the end of life, designed for healthcare professionals, patients and those important to them. The majority of resources focused on healthcare professionals and patients as target audiences. A minority were aimed at specific populations (e.g. patients in specific disease groups). Advance care planning was the most frequently mentioned domain in the resources catalogue. Amongst interactive resources, themes arose including: duplication; the absence of comparator tools in most domains; inconsistency of approaches between guidance documents; practitioner workflow systems integration; and incentivisation.

### Strengths and limitations—Search approach considerations

The initial search covered key organisations suggested by research team members, who had knowledge and experience of this area of care provision. This, combined with additional systematic internet searches, should ensure that the catalogue contains the most prominent resources for clinicians and researchers. Furthermore, we have clearly documented our approach and used tools which are freely accessible (see supplementary materials). It is therefore a reproducible approach that can be easily updated in the future.

There are several limitations. We limited the search to the top 50 results per search term; the catalogue therefore reflects the most visible resources, rather than being comprehensive. This may apply in particular to point-of-care tools, which may not have a strong online presence. We considered only tools developed by UK-based organisations, or claiming relevance to UK practice. Search engines such as Google are highly responsive to changes in search engine optimisation algorithms, and user behaviour; the search strategy might not yield the same results for different users, even after a short period of time. The resources themselves are also updated regularly, and it is not always easy to be certain that one is looking at the latest version of a given resource. Beyond that, some resources contained links to other resources which had expired or referenced outdated practices; this catalogue will similarly become outdated over time.

### Themes arising from interactive resources

#### Duplication and overlap

Duplication (where two or more tools performed the same function) and cross-referencing across prognostic identification tools and advance care plans was evident. Arguably, NICE itself contributes to this duplication by suggesting that healthcare providers develop their own systems for identifying patients entering their last year of life “using tools such as the Gold Standards Framework, the Amber Care Bundle or the Supportive and Palliative Care Indicators Tool [SPICT]” [1]. These layers of cross-referencing risk making the landscape of resources harder to navigate for healthcare professionals. In the absence of a comprehensive evidence base for a single best patient identification tool, it is possible that the variety of tools identified by NICE will contribute to variability in identification of patients entering their last year of life, and make it harder for providers to adapt to the latest best practice [28]. For example, the GSF and the Amber Care Bundle both suggest using the ‘surprise question’, a screening tool for identifying patients at the end of their life, while the SPICT does not [9, 10, 13]. A 2017 systematic review investigating the effectiveness of the surprise question found it varied from poor to reasonable in different studies and suggested that it be incorporated with other prognostic identification tools [29]. More generally, this variation in approaches suggested may reflect an under-developed evidence base, and associated uncertainty about what works best for people approaching end of life, though it is also likely that preferences will vary and a ‘one-size-fits-all’ approach may be inappropriate.

The landscape is rendered even noisier by the intersection of palliative care with a variety of diseases, several of which have their own disease-specific guidance relating to end of life. For example, it may be unclear whether the PiPS-B prognosticator or GSF would be the most appropriate tool to assess the status of a cancer patient [9, 11]. Given the variety of tools available, Healthcare Improvement Scotland’s ​​Palliative Care Identification Tools Comparator provides an important contribution [14]. It gives side-by-side comparisons of different features offered by prognostic tools. This could help clinicians in service provision, and this approach is ripe for transfer across to other domains where there is duplication across resources (e.g. advance care planning documentation).

More primary research investigating the difficulties for healthcare professionals in navigating the resource landscape would further elucidate how tools should be developed in the future. In Spain, a retrospective cohort study was recently completed demonstrating the effectiveness of the NECPAL prognostic tool [30]. It is important that the results of such studies are included in future comparator tools to help clinicians identify the most appropriate resources for their patients.

#### Digital interface and reliability

The task of selecting the most appropriate prognostic tool might be simplified if the day-to-day technology used by clinicians incorporated good practice for these assessments. One such digital tool identified in our internet search was the EARLY search tool, published by Coordinate My Care (NHS) and run on primary care electronic systems [31]. In the USA, a similar technology is being piloted via electronic health records, using a deep learning neural network approach to prioritise patients who are likely to require palliative review [32]. Importantly, the machine learning model has built-in explainability features to allow clinicians to see the reasons why a patient has been selected for palliative review. Making use of these types of technologies could alleviate some of the administrative and selection burden facing healthcare providers seeking to deliver best practice in identifying patients entering their final year of life.

Despite the resources for advance care planning being highly variable, electronic palliative care coordination systems (EPaCCS) designed to share patient preferences between health and social care providers are increasingly being adopted in the UK healthcare system [33]. However, a growing body of evidence suggests that their benefits are limited due to the additional administrative burden they place in filling out the documentation, and the lack of interoperability with other commonly used electronic health care systems [33]. In some cases, this has led to EPaCCS systems not being accessed because of low confidence in the data quality held within the system. Petrova and colleagues have summarised the importance of managerial, financial and cultural drivers for the success and adoption of such systems [34]. The NHS app, which has been widely downloaded throughout the COVID-19 pandemic, now accruing over 22 million users, integrates patient records and may present a vehicle for overcoming some of the administrative challenges associated with siloed EPaCCS systems [35].

#### Incentives

Finally, incentives to use interactive resources could also be an enabler of best-practice adoption in end-of-life care. The Daffodil Standards resource was the only interactive resource we found with built-in incentivisation for practices to adopt their guidelines. It is noteworthy that the incentive in the resource is not financial but rather focuses on demonstrating quality to practitioners, regulators, patients and others. This may be due to concerns about public opinion on the topic of financially incentivising end-of-life care, as demonstrated by the Liverpool Care Pathway [36]. The Daffodil Standards were piloted in autumn 2018, and rigorous analysis of how practices are adopting them, and the effect of their adoption, is not yet available [37]. In the absence of clear understanding of the effectiveness and unintended consequences of both interventions and incentive systems, incentives should be approached with caution, in order to garner public confidence as well as scale-up the adoption of best practice in end-of-life care.

### Moving forward

#### Analyses

The inductive characterisation presented in this paper captures themes and features that could contribute to the efficacy of resources aimed at patients, doctors and family members related to EoLT + C. Others have attempted similar projects in related fields. In surgery, for example, researchers have collated and analysed patient educational resources by systematically scoring their quality and readability [38, 39]. Another study has attempted to categorise and assess 20 patient educational materials related to advanced care planning [40]. Their analysis led to recommendations on which educational tool to use depending on patient readiness. This style of analysis based on our repository of 246 resources could be explored for an even more tailored recommendation engine that incorporates resources for doctors, patients and family members.

#### Maintenance and further development

We present a starting point for a methodology and dataset for cataloguing end of life care resources; in an active field of research and practice, these resources will evolve over time, and so this is not an optimal and final catalogue. Regular maintenance, data enhancement and quality assurance should be an iterative process if such a resource is to remain useful over time. This would be best achieved through institutional investment and collaboration, which may be merited if the catalogue proves useful to physicians, researchers, patients and those close to them. The long-term future of this type of effort could potentially involve automation of searches at regular intervals, combined with expert labelling of subjective resource categories/features. This would improve labour utilisation, visibility, and ultimately end-of-life experiences in our communities.

## Conclusions

We present a replicable and scalable approach for cataloguing and characterising resources surrounding treatment and care at the end of life which are available in a specific country. Our labelled catalogue of almost 250 resources accessible in the UK is now internationally available for healthcare teams, patients and those important to them. The number of resources available suggests that the large-scale problems of implementing best-practice guidance are not due to shortage of information or tools, but lack of adoption at the provider level. However, this in turn may be due in part to the range and overlap of those resources, and the absence of curation of what is available, or standardised recommendations for what to use. Our study presents a first step towards addressing this situation, and may be of benefit to those in other countries who are developing their own resources. In addition to making the catalogue accessible for clinicians and researchers alike, our insights into the characteristics of the resources available may contribute to the wider discussion on increasing the use of advance care planning good practice in end-of-life care—a major challenge in health systems around the world.

## Supplementary Information

**Additional file 1.** 

**Additional file 2.**

## Data Availability

All data generated or analysed during this study are included in this published article [and its supplementary information files].

## Abbreviations

CQC: Care Quality Commission
EoLT + C: End-of-Life Treatment and Care
EPaCCS: Electronic Palliative Care Coordination Systems
GSF: Gold Standards Framework
NHS: National Health Service
NICE: National Institute for Health and Care Excellence
SPICT: Supportive and Palliative Care Indicators Tool
