# Supplementary material for: Towards cataloguing and characterising advance care planning and end-of-life care resources
Source: BMC Palliat Care. 2022 Nov 29;21:211. doi: 10.1186/s12904-022-01102-3 (PMC9706845; doi:10.1186/s12904-022-01102-3)
Supplement: Supplementary file 1 — Additional file 1. [file 12904_2022_1102_MOESM1_ESM.docx]

**Appendix 1: Search terms methodology for resource catalogue**

For every organisation, a Google search was begun with ‘site:’ – ensuring that there is no capitalisation, then website name from the organisation list (without www, no space after : ). Search terms "advance care" OR "end of life" OR “palliative” (space between website name and first search term) were then added, ensuring phrases were marked with quotation marks and OR was capitalised. An example is as follows: site:ageuk.org.uk "advance care" OR "end of life" OR “palliative”

The first 20 results for each search were screened by clicking on the link for each result and checking whether the material accessed matched the inclusion criteria. The initial landing page and any potentially relevant links on that page were screened and added to the catalogue where appropriate.

For finding resources outside of the list of organisations set by the team,Google searches were carried out using a variety of combinations of the following terms by appending the words "Resources", "Information", "Toolkit", "Advice", "Guidance" to the phrases "End of Life Care", "Palliative Care", and "Advance Care Plan". For the sake of reproducibility, the individual explicit searches used were. :

“End of life care resources”, “End of life care information”, “End of life care toolkit”, “End of life care advice”, “End of life care guidance”, “Palliative care resources”, “Palliative care information”, “Palliative care toolkit”, “Advance care plan resources”, “Advance care plan information”, and “Advance care plan toolkit”

Appending “advice” and “guidance” was discontinued for “Palliative Care” and “Advance Care Plan” because those words did not yield additional results when appended to “End of Life Care”.

The first 50 hits for each search were screened by clicking the link and determining whether the material accessed met inclusion criteria and whether it had already been captured in the catalogue. Relevant additional resources were added to the catalogue.
