# Supplementary material for: Towards cataloguing and characterising advance care planning and end-of-life care resources
Source: BMC Palliat Care. 2022 Nov 29;21:211. doi: 10.1186/s12904-022-01102-3 (PMC9706845; doi:10.1186/s12904-022-01102-3)
Supplement: Supplementary file 2 — Additional file 2. [file 12904_2022_1102_MOESM2_ESM.docx]

**Appendix 2: Link to catalogue on google sheets**

<https://docs.google.com/spreadsheets/d/1q0e7hDPSaSlcV91OfndKFzSFeZcTTEjd/edit?usp=sharing&ouid=100362785142244297437&rtpof=true&sd=true>
